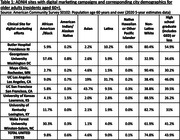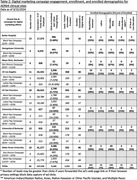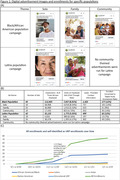# Digital marketing for increasing participant diversity in ADNI4 – early insights from initial rollout

**DOI:** 10.1002/alz.095041

**Published:** 2025-01-09

**Authors:** Catherine C. Conti, Hannatu Amaza, Melanie J. Miller, Mai Seng Thao, Derek Flenniken, Winnie Kwang, Miriam T. Ashford, Juliet Fockler, Diana Truran‐Sacrey, Joseph Strong, Jennefer Sorce, Roxanne Alaniz, Michael W. Weiner, Ozioma C Okonkwo, Monica Rivera Mindt, Rachel L. Nosheny

**Affiliations:** ^1^ Northern California Institute for Research and Education (NCIRE), San Francisco, CA USA; ^2^ San Francisco Veterans Affairs Medical Center, San Francisco, CA USA; ^3^ Wisconsin Alzheimer’s Disease Research Center, School of Medicine and Public Health, University of Wisconsin‐Madison, Madison, WI USA; ^4^ University of California, San Francisco, San Francisco, CA USA; ^5^ Veterans Affairs Medical Center, San Francisco, CA USA; ^6^ Alaniz Marketing, Novato, CA USA; ^7^ Icahn School of Medicine, Mount Sinai Hospital, New York, NY USA; ^8^ Fordham University, New York, NY USA

## Abstract

**Background:**

The Alzheimer’s Disease Neuroimaging Initiative (ADNI4), whose goal is to validate biomarkers for AD clinical trials, aims to increase participation of individuals from historically underrepresented populations (URPs) so results are more generalizable. ADNI uses digital marketing to recruit and enroll older adults into a digital cohort, for screening into in‐clinic ADNI. Challenges and insights from initial marketing efforts, including enrollment rates, local demographics, and effectiveness at enrolling URPs are presented.

**Methods:**

We employed a culturally‐informed marketing approach developed in partnership with ADNI’s Community Scientific Partnership Board and marketing firm, including digital campaigns (Meta ads, websites, and emails) designed using themes aimed to resonate with Black and Latinx communities (solo, family, community; Fig. 1). Varying ads run simultaneously to compare effectiveness. Ads direct to study websites, registration pages, and consent to join a digital cohort. Ads run in geographies based on clinical site readiness to enroll participants.

**Result:**

Most initial sites to utilize digital marketing (Universities of Kansas, Kentucky, and Mayo Clinic) were in areas of lower ethnocultural diversity compared to other ADNI sites (Table 1). Three months into recruitment, we shifted to optimizing ads in an area with higher ethnocultural diversity (UKentucky) to maximize data on highest enrolling ads (Tables 1&2). Additional digital recruitment began for sites in areas with higher ethnocultural diversity (UCSF, Wake Forest; Table 1). We subsequently saw a 317% increase in enrollment of Black older adults, with no significant improvement for other URPs (Fig. 1c). Following campaign launches (Butler, Georgetown) further supported our campaigns’ ability to enroll Black adults (Table 2). Initial results suggest “solo” and “community” themed ads yield highest enrollments in Black population campaigns. (Fig. 1b). Despite running in areas with large Latinx populations (UCLA), ADNI4’s Latinx campaigns were generally not obtaining enrollment rates comparable to our Black population campaigns (Tables 1&2).

**Conclusion:**

Our early successes in recruitment of older Black adults (43% of digital cohort enrollments) strongly demonstrates the feasibility of culturally‐informed, site‐specific digital marketing to specific URPs for AD clinical studies. More work is needed to increase inclusion of Latinx adults (7% of enrollments) and other URPs in ADNI4’s digital cohort.